# Hsv-1 Endocytic Entry into a Human Oligodendrocytic Cell Line Is Mediated by Clathrin and Dynamin but Not Caveolin

**DOI:** 10.3390/v12070734

**Published:** 2020-07-07

**Authors:** Beatriz Praena, Raquel Bello-Morales, José Antonio López-Guerrero

**Affiliations:** 1 Departamento de Biología Molecular, Universidad Autónoma de Madrid, Cantoblanco, 28049 Madrid, Spain; Raquel.bello-morales@uam.es (R.B.-M.); jal@cbm.csic.es (J.A.L.-G.); 2 Centro de Biología Molecular Severo Ochoa, CSIC-UAM, Cantoblanco, 28049 Madrid, Spain

**Keywords:** HSV-1, endocytosis, clathrin, dynamin, oligodendrocyte

## Abstract

Endocytosis is a pathway used by viruses to enter cells that can be classified based on the proteins involved, such as dynamin, clathrin or caveolin. Although the entry of herpes simplex type 1 (HSV-1) by endocytosis has been documented in different cell types, its dependence on clathrin has not been described whereas its dependence on dynamin has been shown according to the cell line used. The present work shows how clathrin-mediated endocytosis (CME) is one way that HSV-1 infects the human oligodendroglial (HOG) cell line. Partial dynamin inhibition using dynasore revealed a relationship between decrease of infection and dynamin inhibition, measured by viral titration and immunoblot. Co-localization between dynamin and HSV-1 was verified by immunofluorescence at the moment of viral entry into the cell. Inhibition by chlorpromazine revealed that viral progeny also decreased when clathrin was partially inhibited in our cell line. RT-qPCR of immediately early viral genes, specific entry assays and electron microscopy all confirmed clathrin’s participation in HSV-1 entry into HOG cells. In contrast, caveolin entry assays showed no effect on the entry of this virus. Therefore, our results suggest the participation of dynamin and clathrin during endocytosis of HSV-1 in HOG cells.

## 1. Introduction

Herpes simplex virus type 1 (HSV-1) is a ubiquitous infectious agent that can establish latency in the sensory ganglia of the peripheral nervous system and affects around 90% of the adult population in industrialized countries [1]. Although the majority of individuals with HSV-1 infection do not develop any disease, common symptoms of oral herpes include painful blisters or open ulcers in/or around the mouth known as cold sores. In rare cases, HSV-1 may also cause more severe complications such as encephalitis or keratitis following eye infection [2]. Thus, molecular studies of this infectious agent are of vital importance for the understanding of virus–cell interactions during the viral infection cycle, including the entry step. In fact, many details of HSV-1 cellular entry remain to be unraveled, even though *Herpesviridae* are one of the most studied families of viruses.

HSV-1 is able to enter several host cell types using different strategies, an ability that might be due to the number of viral glycoproteins involved, the existence of multiple alternative cell receptors, and the varied strategies and mechanisms available [3]. Depending on the cell model, HSV-1 can enter via pH-independent fusion between viral lipid envelopes and host membranes, phagocytosis or endocytosis, or a combination of these routes [4,5]. In all cases, entry is mediated through the interaction of viral glycoproteins D (gD), B (gB), C (gC) and H/L (gH/L) with different cellular receptors [6]. These receptors are grouped according to their affinity to viral glycoproteins: gB and gC recognize and interact with heparan sulfate proteoglycans (HSPGs), whereas gD can recognize herpes virus entry mediator (HVEM), Nectin-1 and 2, or 3-O-sulfated heparan sulfate (3-OH-HS) [7].

Endocytosis begins with a weak interaction between various binding factors of the cell membrane and the envelope of HSV-1, followed by a specific recognition between one or more cell receptors and the viral glycoproteins gD and gH/gL [8]. Multivalent binding leads to a grouping of receptors which can give rise to an association with lipid domains and activation of several signaling pathways [9]. In particular during HSV-1 infection, the first step during adsorption is the recognition of heparan sulfate (HS) moieties by viral gB or gC [10].

Adsorption triggers internalization through endocytosis, which may involve clathrin-mediated endocytosis (CME), macropinocytosis, caveolin- or flotillin-mediated endocytosis, or a variety of other poorly characterized mechanisms (such as lipid raft-dependent mechanisms) [11]. CME is one of the most well studied and characterized pathways and is a commonly used mechanism for viral entry into cells. Furthermore, in response to different cellular signals, cytoplasmic clathrin molecules are recruited to the membrane where the entry process takes place [12], forming clathrin-coated pits (CCPs) which induce a curvature in the membrane, leading to vesicle formation.

Dynamin is a GTPase required for constriction of the vesicle and its excision into the cell cytoplasm. The implication of dynamin in viral endocytosis has been widely accepted [13,14,15], and though there exist endocytic pathways that lack this protein, it is crucial in CME. This dynamin belongs to the fission group, which is cytosolic proteins that are recruited peripherally and reversibly to membranes in the endocytosis pathway [16]. Another common process ending with the formation of a primary vesicle involves lipid rafts and cholesterol as well as phosphatases and kinases [9]. Lipid rafts present in the cell form specialized membrane microdomains enriched in cholesterol and sphingolipids that can alter the fluidity of the membrane, receptor clustering and the assembly of signaling molecules [17]. In the clathrin-independent case of lipid raft-dependent endocytosis [18], dynamin may or may not be necessary, although, in some cases, caveolin can be part of this alternative entry process (caveolin-mediated endocytosis) [19]. In fact, caveolin is a transmembrane protein that clusters in lipid rafts, so unlike clathrin, it does not need to be recruited from the cellular cytoplasm [20]. 

In this work, we have studied the roles of clathrin, caveolin and dynamin in the endocytic entry of HSV-1 into the established human oligodendroglial (HOG) cell line. Dynamin and clathrin might play significant roles in endocytosis, while caveolin does not appear to be involved in the process. 

## 2. Materials and Methods 

### 2.1. Cell Culture 

The human HOG cell line [21] was established from a surgically removed human oligodendroglioma and kindly provided by A. T. Campagnoni (University of California, Los Angeles, USA). Cells were cultured in growth medium (GM) containing low-glucose Dulbecco’s Modified Eagle Medium (DMEM) supplemented with 5% fetal bovine serum (FBS), penicillin (50 U/mL) and streptomycin (50 μg/mL) at 37 °C in a humidified atmosphere of 5% CO_2_. To induce differentiation, cells were cultured in serum-free differentiation medium (DM), containing low-glucose DMEM supplemented with penicillin (50 U/mL) and streptomycin (50 μg/mL), 50 µg/mL apo-transferrin (T203), 0.5 mg/L insulin (I9278), 30 nM triiodothyronine (T3), 30 nM sodium selenite and 16.1 mg/L putrescine (P5780). Cells cultured in this medium were also treated with 0.5 mM Dibutyryl-cAMP (dbcAMP) (D0627) (reagents from Sigma Chemical Co., St. Louis, MO, USA). Vero cells were kindly provided by Enrique Tabarés (Universidad Autónoma de Madrid) and cultured in GM.

### 2.2. Viruses

Wild-type (wt) HSV-1 (F strain) (GenBank accession number for the DNA genome sequence is GU734771): HSV-1 K26-GFP was obtained by fusing green fluorescent protein (GFP) to the HSV-1 capsid protein VP26 [22] (a kind gift from. Desai; Johns Hopkins University, Baltimore, USA). K26-GFP and wild-type HSV-1 viruses were propagated and titrated on Vero cells. The HSV-gL86 virus, which expresses β-galactosidase, was a kind gift from. R. Longnecker (Northwestern University, Chicago, USA). HSV-gL86, a mutant in which the *E. coli* lacZ gene replaces part of the gL open reading frame, was propagated and titered on gL-expressing Vero cells 79VB4 [23].

### 2.3. Antibodies and Reagents

Dynasore (D7693), chlorpromazine (C8138), nystatin (N6261), *o*-nitrophenyl-β-D-galactopyranoside (ONPG), low-glucose DMEM and FBS were purchased from Sigma Chemical Co. (St. Louis, MO, USA).

Rabbit anti-dynamin 2 polyclonal antibody and peroxidase-conjugated anti-rabbit immunoglobulin G (IgG) were from Invitrogen (CA, USA), and rabbit anti-HSV-1 polyclonal antibody was from Dako (CA, USA). Alexa Fluor 555 secondary antibody was from Molecular Probes (Eugene, OR, USA). To-Pro-3 was from ThermoFisher (MA, USA). Mowiol was from Calbiochem (Merck Chemicals, Darmstadt, Germany), and human transferrin (Trf) CF^®^543 and cholera toxin subunit B (CT-B) CF^®^543 conjugates were purchased from Biotum (San Francisco, CA, USA).

### 2.4. Cell Viability Assay 

To evaluate the potential cytotoxic effect of the specific inhibitors of dynamin (dynasore), CME (chlorpromazine) and caveolin (nystatin) in HOG cells, an (3-(4,5-dimethylthiazol-2-yl)-2,5-diphenyltetrazolium bromide) tetrazolium (MTT) assay (Roche-11465007001) was performed. Non-confluent monolayers of HOG cells plated in 96-well tissue culture dishes and cultured in DM were incubated for 2 h with different concentrations between 10 μM and 100 μM of each inhibitor. Then, cells were incubated with a final concentration of 0.5 mg/mL of MTT in a humidified atmosphere for 4 h, and formazan crystals were solubilized in 0.01 M HCl with 10% SDS. The resulting colored solution was quantified using a scanning multi-well spectrophotometer (ELISA reader), measuring the absorbance of formazan at 595 nm.

### 2.5. Immunofluorescence Microscopy

Cells were plated in 24-well plates on round coverslips (1.2 × 10^5^ cells/well) and cultured in DM for 24 h, fixed in 4% paraformaldehyde (PFA) for 20 min and rinsed with phosphate buffered saline (PBS). Cells were then permeabilized with 0.2% Triton X-100, rinsed and incubated for 30 min with 3% bovine serum albumin in PBS, adding 10% human serum when necessary (to block the HSV-1-induced IgG Fc receptors). For a labelled immunofluorescence analysis, cells were incubated for 1 h at room temperature with the appropriate primary antibodies, rinsed several times and incubated at room temperature for 30 min with the relevant fluorescent secondary antibodies. Dilutions of anti-dynamin 2 were prepared in the presence of 10% human serum. Controls to assess labelling specificity included incubations with control primary antibodies or omission of the primary antibodies [24]. 

After thorough washing, coverslips were mounted in Mowiol. Images were obtained using an LSM510 META system (Carl Zeiss, Jena, Germany) coupled to an inverted Axiovert 200 microscope (Carl Zeiss, Jena, Germany). Cholera toxin assay images were obtained using an sCMOS monochrome (Zeiss) coupled to an inverted Axiovert200. Processing of confocal images was performed by FIJI-ImageJ software (ImageJ2).

### 2.6. β-Galactosidase Assay

For this aim, cells were plated in 96-well plates and treated with nontoxic concentrations of the compounds. Subsequently, cultures were infected with HSV-1 gL86, a recombinant virus which expresses β-galactosidase upon entry into the cells, at an multiplicity of infection (m.o.i.) of 5. The substrate ONPG was added, and the colorimetric reaction was tracked at 410 nm in a microplate reader.

### 2.7. Electron Microscopy

To study the viral entry by endocytosis, electron microscopy (EM) analysis was performed. HOG cells were plated on P24 dishes (1.2 × 105 cells/well) and cultured in DM. Cells then were infected or mock-infected with HSV-1 at an m.o.i. of 100 for 30 min at 4 °C and then for 20 min at 37 °C in a humidified 5% CO_2_ incubator. HOG cells were fixed in 4% PFA and 2% glutaraldehyde (GTA) in 0.1 M phosphate buffer and processed for conventional TEM, as described previously [25].

Grids were examined in a JEOL JEM-1010 transmission electron microscope (Jeol, Tokyo, Japan), and all images were recorded with a TemCam-F416 (4K×4K) digital camera from Tietz Video (Gauting, Germany) and Image Processing Systems (TVIPS; Martinsried, Germany).

### 2.8. Immunoblot Analysis

Samples containing equal number of cells were subjected to SDS-PAGE in 12% acrylamide gels under reducing conditions and transferred to Immobilon-P membranes (Millipore, Darmstadt, Germany). After blocking with 5% nonfat dry milk and 0.05% Tween 20 in PBS, blots were incubated for 1 h at room temperature with a rabbit anti-HSV-1 polyclonal antibody. After washing several times with 0.05% Tween 20 in PBS, blots were incubated for 1 h with the secondary antibodies coupled to horseradish peroxidase, washed extensively and developed using an enhanced chemiluminescence western blotting kit (ECL, Amersham, Little Chalfont, UK).

### 2.9. Real-Time Quantitative RT-PCR Assay

Total RNA from quadruplicate samples of HOG cells cultured in 60 mm dishes under differentiation condition and infected at an m.o.i. of 1 with HSV-1 at 3 h postinfection (p.i.) was extracted using RNeasy Mini kit (Qiagen, Valencia, CA, USA). HOG cells had been previously pretreated with chlorpromazine, maintaining the compound throughout infection. Later, RNA was quantified in a Nanodrop ND-1000 spectrophotometer (Thermo Fisher Scientific, MA, USA), testing its integrity with an Agilent 2100 Bioanalyzer (Agilent Technologies, Santa Clara, CA, USA). All samples showed 260/280 ratio values around 2, corresponding to pure RNA. The integrity of RNA samples was scored by Integrity Number (D/R-IN), which showed values between 9.0 and 10.0. Genomic DNA contamination was assessed by amplification of representative samples without real team (RT) enzyme. Real-time quantitative RT-PCR assays were performed as previously described [26,27] with primer sequences targeting the immediate early gene *ICP4* and early gene polymerase (*Pol*) and analyzed by the Genomics Core Facility at Centro de Biología Molecular Severo Ochoa (CSIC-UAM). In order to find the most suitable genes for normalization, the stability of three candidates—*Glyceraldehyde-3-Phosphate Dehydrogenase* (*GAPDH*), *18S* and ubiquitin (*UBQ*)—was analyzed with the NormFinder algorithm. Given its high stability, *GAPDH* was chosen as the most appropriate.

### 2.10. Statistical Analysis

A Student’s *t*-test was performed for independent measures to compare the mean values of each data set, with *p*-values < 0.05 being categorized as significant.

## 3. Results

### 3.1. The Endocytosis Inhibitors Dynasore, Chlorpromazine and Nystatin are Nontoxic in HOG Cells at Established Concentrations

**Figure 1:**
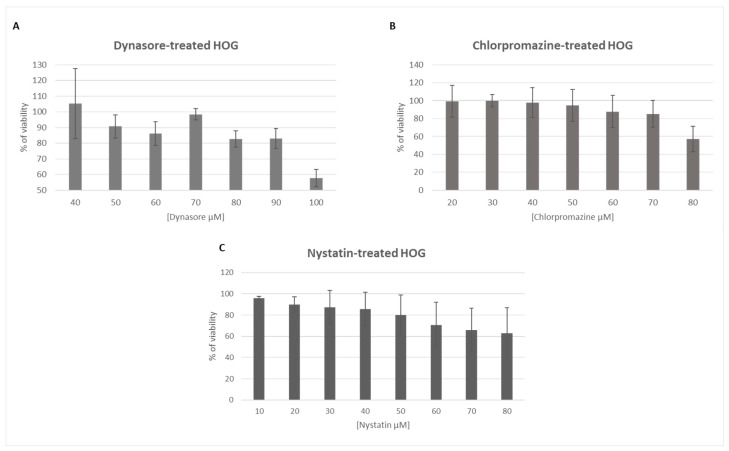
Viability of human oligodendroglial (HOG) cells exposed to specific inhibitors of endocytic pathway components: HOG cells cultured on DM (differentiation medium) were incubated with different concentrations (10–100 μM) of dynasore (**A**), chlorpromazine (**B**) or nystatin (**C**) for 2 h. Cell viability was measured by MTT assay and calculated as a percentage of viable, nontreated cells; columns represent the mean viability ± standard deviation (S.D.) (*n* = 4), after exposure to the drugs.

To study the toxicity of dynasore, chlorpromazine and nystatin, HOG cells were cultured in DM in the presence of different concentrations of each compound for 2 h and cell viability was assessed by an MTT assay. The tolerated concentrations in HOG cells were different depending on the compound (Figure 1).

HOG cells treated with 50 μM dynasore or 40 μM chlorpromazine exhibited more than 90% viability, while cultures treated with 40 μM nystatin had a viability around 85–95%. For further experiments, these highest nontoxic concentrations of each inhibitor were used. 

### 3.2. Endocytosis Assay

Since HSV-1 can reach the cellular cytoplasm by several mechanisms, we wanted to test whether endocytosis could play a significant role during infection of our oligodendrocytic cultures. To this aim, we first needed to establish a suitable method to monitor endocytosis in our cell model. To evaluate dynamin-, clathrin- and caveolin-mediated endocytosis in HOG cells, human Trf CF^®^543, which undergoes clathrin-dependent uptake [28], and CT-B CF^®^543 conjugates, which are taken up by caveolar endocytosis [29], were used as indicators. For this purpose, cells were pretreated for 1 h with the corresponding endocytosis inhibitors and incubated for 1 h on ice with conjugate Trf (5 µg/mL) and conjugate CT-B (1 µg/mL). Finally, cells were maintained for 10 min at 37 °C in a humidified 5% CO_2_ incubator.

**Figure 2:**
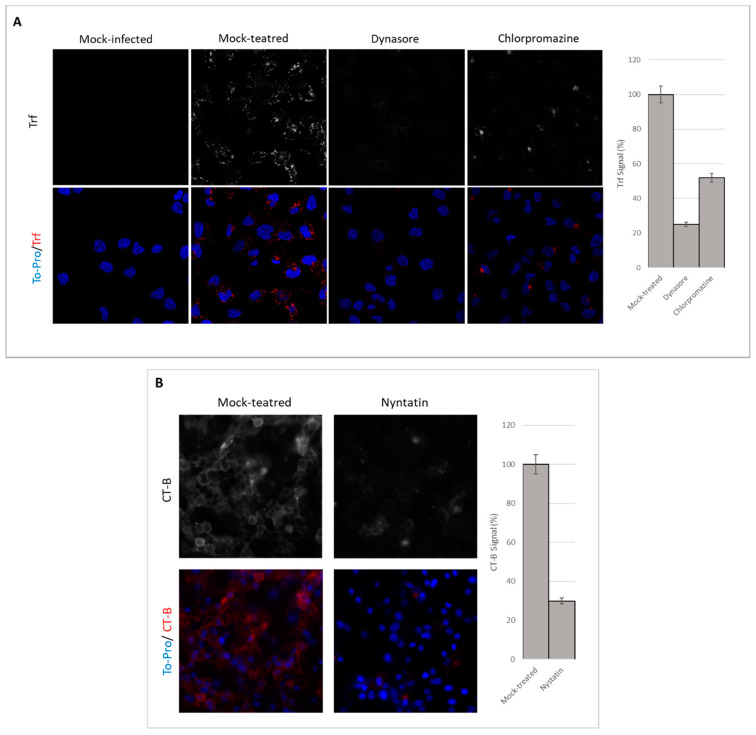
Visualization of endocytosis inhibition: HOG cells were mock-treated or treated with 50 μM dynasore, 40 μM chlorpromazine or 40 μM nystatin for 1 h prior to the internalization of Alexa Fluor 543 Trf (human transferrin) (**A**), which undergoes clathrin-dependent uptake, or CT-B (cholera toxin subunit B) (**B**), which is taken up by caveolar endocytosis, and were analysed by confocal fluorescence microscopy and the images processed by ImageJ2. Nuclei were detected by TO-PRO-3 staining.

Fluorescence microscopy images after inhibitor treatment showed a decrease in Trf and CT-B uptakes, suggesting that dynamin-mediated endocytosis, CME and caveolin-mediated endocytosis had been partially blocked or inhibited (Figure 2). The decrease in Trf absorption in dynasore-treated cells (blockage of dynamin-mediated endocytosis) was drastic compared to that of cultures treated with chlorpromazine and nystatin.

### 3.3. Effect of Endocytosis Inhibition on HSV-1 Infection

**Figure 3:**
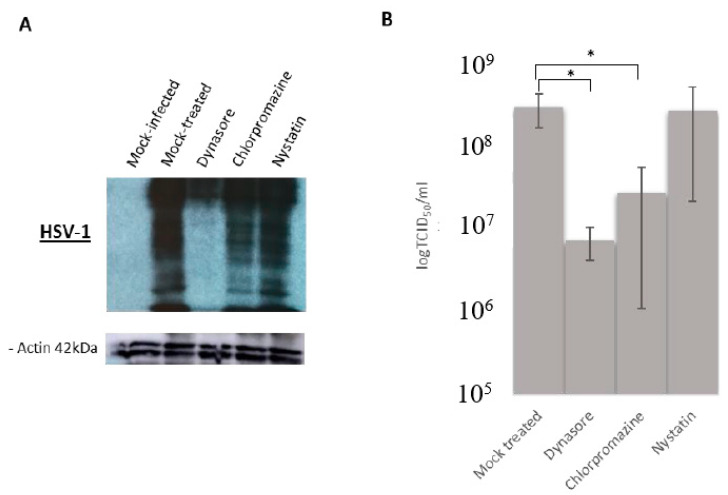
Effect of endocytosis inhibition on herpes simplex type 1 (HSV-1) infection: HOG cells were pretreated with dynamin (50 μM), chlorpromazine (40 μM) or nystatin (40 μM) for 1 h and then infected with HSV-1 at an multiplicity of infection (m.o.i). of 1. (**A**) Lysates from cultured HOG cells were subjected to SDS–PAGE (sodium dodecyl sulphate-polyacrylamide gel electrophoresis) at 16 h p.i. and analysed by immunoblotting with a rabbit anti-HSV-1 polyclonal antibody. (**B**) Viral production was quantified at 16 h p.i. by endpoint dilution (Median Tissue Culture Infectious Dose) TCID50/mL. Values are reported as the mean ± S.D. (*n* = 4), as compared to controls (*p < 0.05).

In order to study whether dynamin-, clathrin- and/or caveolin-mediated endocytosis were involved in HSV-1 entry, HOG cells were treated for 1 h with the inhibitors and then infected with HSV-1 wt for 16 h p.i. Immunoblot assay showed a decrease of viral proteins in cultures of HOG cells pretreated with dynasore or chlorpromazine compared to mock-treated cultures (Figure 3A). Moreover, the decrease was greater in dynasore-inhibited cells compared to the other inhibitors (Figure 3A). Infectious viral titers showed a drastic decrease, around two orders of magnitude, in HOG cells treated with dynasore (Figure 3B) and a less dramatic but still significant decrease in cells treated with chlorpromazine (Figure 3B). Nystatin had no significant effect on viral production, indicating that caveolin may not play a role in viral internalization.

### 3.4. Role of Dynamin in HSV-1 Infections 

**Figure 4:**
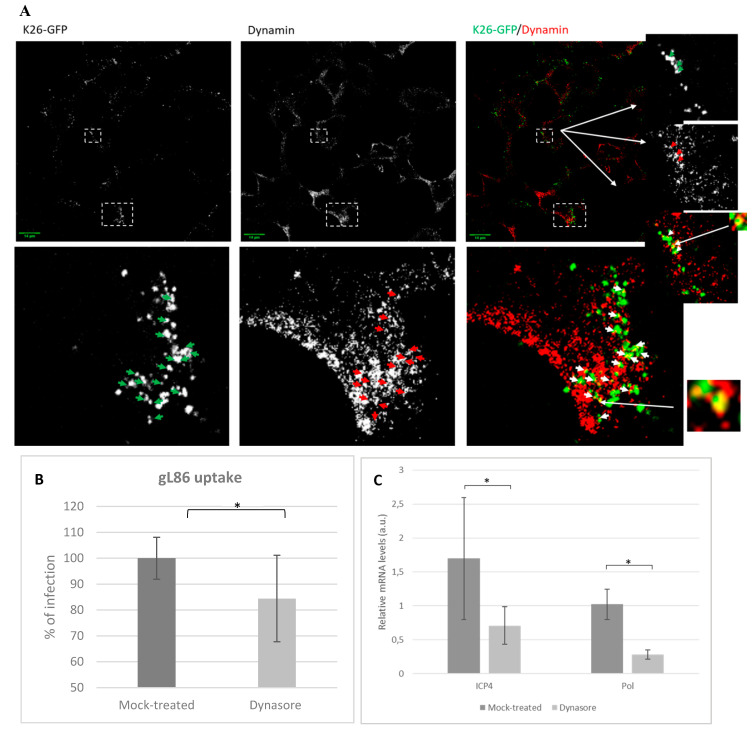
Infection of HOG cells treated with the dynamin inhibitor: (**A**) Co-localization of dynamin with HSV-1. HOG cells infected at an m.o.i. of 1 with K26-GFP were fixed at 15 min p.i. and processed for indirect immunofluorescence confocal microscopy with an anti-dynamin monoclonal antibody followed by an anti-mouse Alexa 555 secondary antibody. Images correspond to 0.8 μm confocal slices. Arrows point to possible co-localization events of virions with dynamin. Dashed squares show the location of enlarged images (bottom and right) on the confocal fields. (**B**) Confluent monolayers of HOG cells treated or mock-treated with dynasore 50 μM were infected with a recombinant HSV-1 (KOS) gL86 at an m.o.i. of 5. After 6 h p.i., the β-galactosidase activity (absorbance at 410 nm) was analysed in a microplate reader. The bars show the average infection based on the optical density (OD). (**C**) RT-qPCR of mRNAs: Bars represent relative mRNA levels corresponding to ICP4 and polymerase (Pol) extracted at 3 h p.i. from HSV-1 wild-type-infected HOG cells treated or mock-treated with dynasore 50 μM. (a.u., arbitrary units.) Values are reported as the mean ± S.D. (*n* = 3), relative to uninfected controls (* *p* < 0.05).

To further study the role of dynamin in HSV-1 entry, HOG cultures infected with the recombinant K26-GFP virus were analysed by confocal fluorescence microscopy. Our results showed the possible partial co-localization between dynamin and virions (Figure 4A), supporting a role for dynamin-mediated endocytosis in viral entry. After adsorption for 30 min at 4 °C followed by 20 min incubation at 37 °C, co-localization of fluorescence signals could be detected near the cellular membrane, suggesting the feasible interaction of virions and dynamin.

To investigate the role of dynamin in viral entry, a gL86 assay was performed. HOG cells were plated in 96-well plates and treated with dynasore 50 μM, a specific dynamin inhibitor. Subsequently, cells were infected at an m.o.i. of 5 with HSV-1 (KOS) gL86. At 6 h p.i., we analyzed the β-galactosidase expression using a microplate reader and represented the results as the maximum percentage of untreated infected cells. In accordance with our previous data, the results showed a decrease in optical density (OD) in dynasore-treated cells compared to the mock-treated cultures at 6 h p.i. (Figure 4B).

To confirm that dynamin plays a role during HSV-1 infections, dynasore-treated cells were infected with HSV-1 wt and the expression of the viral immediate early gene *ICP4* and early gene *Pol* were quantified at 3 h p.i. by RT-qPCR (Figure 4C). Results revealed a significant decrease in experession of both *ICP4* and *Pol* genes (Figure 4C) compared to controls.

### 3.5. Role of Clathrin in Viral Infection by Endocytosis 

**Figure 5:**
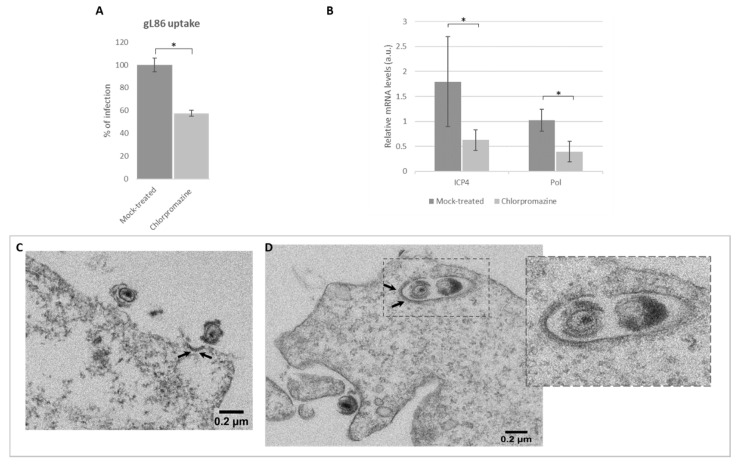
Effect of clathrin-mediated endocytosis (CME) inhibition on HSV-1 entry: (**A**) Confluent monolayers of HOG cells plated on 96-well tissue culture treated or mock-treated with chlorpromazine 40 μM were infected with a recombinant HSV-1 (KOS) gL86 at an m.o.i. of 5. After 6 h p.i., the β-galactosidase activity (absorbance at 410 nm) was analysed in a microplate reader. The bars show the average infection based on the optical density (OD). (**B**) RT-qPCR of mRNAs. Bars represent relative mRNA levels corresponding to ICP4 and polymerase (Pol) extracted at 3 h p.i. from HSV-1 wt-infected HOG cells treated or mock-treated with chlorpromazine 40 μM. (a.u., arbitrary units.) Values are reported as the mean ± S.D. (*n* = 8), relative to uninfected controls (* *p* < 0.05). HOG cells grown in DM were plated in 24-well tissue culture dishes and infected with HSV-1 wild type at an m.o.i. of 100. After 20 min, HOG cells were fixed and processed for observation using electron microscopy (EM). Arrows point to (**C**) a clathrin-coated pit (CCP) and (**D**) a clathrin-coated vesicle (CCV). The inset is an enlarged view of the confocal field.

Once the toxicity and the effect of the inhibitors on viral production were ascertained, we performed an entry assay to confirm the role of clathrin in viral entry. HOG cells were plated in 96-well plates and treated with chlorpromazine, a specific CME inhibitor. Subsequently, cells were infected at an m.o.i. of 5 with HSV-1 (KOS) gL86. At 6 h p.i., we analyzed the β-galactosidase expression using a microplate reader and represented the results as the maximum percentage of untreated infected cells. In accordance with our previous data, the results showed a decrease in OD in chlorpromazine-treated cells compared to the mock-treated cultures (decrease from 100% to 60%) at 6 h p.i. (Figure 5A).

To further confirm that HSV-1 uses CME for entry, the expression of the viral immediate early gene *ICP4* and early gene *Pol* was quantified at 3 h p.i. by RT-qPCR. For this purpose, HOG cells were treated with chlorpromazine and infected with HSV-1 wt. Results revealed a significant decrease of mRNA synthesis in both *ICP4* and *Pol* genes (Figure 5B) compared to controls. 

To perform ultrastructural analysis, EM assay was performed. Observation of clathrin in EM does not require immunolabeling, since the protein net surrounding the endocytic vesicles is naturally electrodense and can be detected directly by negative staining. Results showed virions near electrodense zones compatible with an incipient CCP (Figure 5C) and virions enclosed in endocytic clathrin-coated vesicles (CCV; Figure 5D). These results suggest that clathrin plays a significant role in HSV-1-mediated endocytosis.

### 3.6. HSV-1 Entry by Lipid Raft-Mediated Endocytosis 

**Figure 6:**
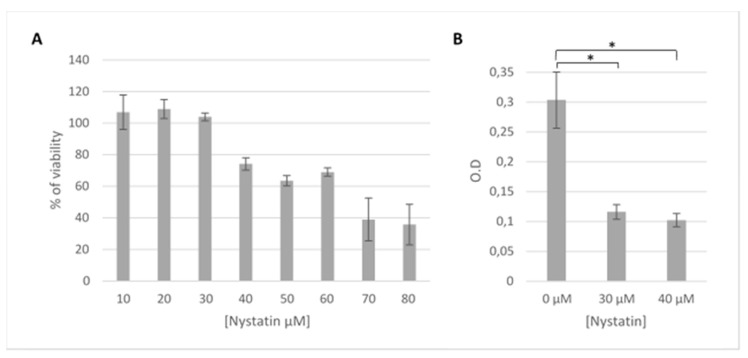
Effect of nystatin on HSV-1 entry: (**A**) HOG cells in 96-well plates were incubated with different concentrations of nystatin (ranging from 10–80 μM) for 24 h. Cell viability was measured by MTT assay and calculated as a percentage of viable nontreated cells; columns represent the mean viability ± S.D. (*n* = 4), after exposure to the drug. (**B**) Confluent monolayers of HOG cells in 96-well plates treated or mock-treated with nystatin (30 or 40 μM) were infected with a recombinant HSV-1 (KOS) gL86 at an m.o.i. of 5. After 6 h p.i., β-galactosidase activity was indirectly measured by reading absorbance at 410 nm in a microplate reader. The bars show the OD, a measure of HSV-1 gL86 virus entry based on its β-galactosidase expression (* *p* < 0.05).

Another possible mechanism for viral entry involves lipid rafts in the cell membrane of host cells. In order to ascertain HSV-1’s usage of lipid raft-mediated endocytosis, HOG cells were treated with nystatin for 24 h, as described previously [30,31]. First, an MTT assay with nystatin maintained for 24 h was performed in contrast with the first MTT assay, which was performed and maintained for 2 h, since the compounds were only present during viral adsorption. As shown in Figure 6A, cellular viability of nystatin-treated cultures was 75% in the same conditions as the entry assay. Subsequently, β-galactosidase assays were carried out after infection with HSV-1 gL86; cells were treated with 30 or 40 µM nystatin, which was maintained during adsorption and up to 5 h p.i. The results showed a decrease in viral entry when HOG cells were treated with nystatin compared to nontreated cells (Figure 6B).

## 4. Discussion

Oligodendrocytes (OLs) are the myelin-forming cells of the central nervous system (CNS) [32,33]. The myelin sheath is an electrically insulating layer that surrounds axons in both the central and peripheral nervous systems, allowing saltatory conduction of action potential. Damage in OLs affecting the myelin formation could trigger demyelinating and neurodegenerative diseases [34,35,36]. Previous analysis carried out by our group have shown that OLs are highly susceptible to HSV-1 infection [37], with HOG cells being one of the most suitable established human oligodendroglial cell lines used as a culture model in neurological studies in vitro [38,39,40]. Subsequent works revealed that HOG cells cultured under differentiation conditions became more susceptible to HSV-1 infection [41].

HSV-1 has the ability to enter cells through different pathways [3], with endocytosis being one of the main ones. To date, endocytic entry of HSV-1 has been described in some lines such as fibroblasts and keratinocytes, in which it was independent of clathrin and caveolin [42], and may or may not be dependent on dynamin [14,43]. According to the available evidence, the exact type of entry may vary depending on the cell line [44]. In the current work, we studied the role of endocytosis mediated by clathrin, caveolin and dynamin in the entry of HSV-1 into oligodendrocytic cells.

Dynasore, chlorpromazine and nystatin compounds were selected as specific inhibitors of endocytosis. Dynasore acts as a competitive inhibitor against dynamin GTPase [45], chlorpromazine inhibits the formation of clathrin mesh around the vesicle [46,47,48,49] and the antifungal nystatin acts by sequestering cholesterol from the plasma membrane, therefore decreasing the capacity of caveola formation [50]. 

After choosing nontoxic concentrations (Figure 1), inhibitors of endocytosis were verified by measurement of Trf uptake (mostly by clathrin) and by CT-B uptake (by caveolin) (Figure 2). Trf uptake by dynamin-dependent entry was almost completely inhibited compared to clathrin and caveolin, probably due to its participation in diverse pathways in addition to CME.

Infections after these cell treatments resulted in a decreased viral titer, indicating a relationship between viral entry and endocytosis (Figure 3). Our results suggest that viral entry by fusion at the plasma membrane can also lead to productive infection [5,14,51], since inhibition of endocytosis did not completely blocked infection (Figure 3B). 

Overall, the results suggest that dynamin plays a fundamental role in HSV-1 infection of HOG cells (Figure 3A,B). The relationship between dynamin and viral infection has been verified in other viruses including some polyomaviruses and coronaviruses [52,53] and in HSV-1, depending on the cell type [54,55]. In addition to caveolin-dependent endocytosis and CME, dynamin is known to collaborate in other endocytic pathways [55,56,57]. Thus, inhibition of dynamin not only blocks CME to some extent but also should impact the many alternative routes of entry where it is involved. This would explain the greater decrease in viral infectivity observed in HOG cells treated with dynasore (Figure 3A,B), compared with those treated with chlorpromazine. In fact, in addition to endocytosis, dynamin is involved in other cellular processes such as membrane trafficking, in actin dynamics [58,59,60] or even in vesicles emanating from the Golgi [61,62]. Fluorescence microscopy images showed a partial co-localization between dynamin and HSV-1 in HOG cells (Figure 4A), only during virus entry 20 min after adhesion, thus discarding the processes described above. This result seems to indicate a possible direct interaction between HSV-1 and dynamin during viral entry, which may be related to the endocytic pathway. Additionally, dynasore-treated cells showed a decrease in HSV-1 gL86 infection (Figure 4B), suggesting the involvement of this protein in viral entry. This possible role of dynamin in virus entry was confirmed by RT-PCR in dynasore-treated cultures (Figure 4C), where the expression of the viral immediate early and early genes decreased compared to the nontreated control.

The study of the involvement of clathrin in HSV-1 endocytosis was also carried out using the recombinant virus gL86. The entry of this virus (Figure 5A) was significantly lower in the chlorpromazine-treated cultures than in untreated control cultures. Likewise, chlorpromazine led to a statistically significant decrease in transcription of the immediately early gene *ICP4* and early *Pol* at 3 h p.i. (Figure 5B) (cells infected with HSV-1 wt). In summary, both infection assays and RT-qPCR revealed the possibility that, at least in our oligodendrocytic cell system, HSV-1 uses CME.

CME is the best-described endocytic route and the one most used by various viruses such as adenoviruses [63], enteroviruses [64] and parvoviruses [65] for cell entry. Although this route is preferentially used by medium and small viruses, it has been found that larger viruses such as vesicular stomatitis virus (VSV) can also use it [66,67]. EM clearly showed virions approaching CCPs during attachment (Figure 5C), which is logical since it is known that virions can either produce signals for clathrin recruitment at the moment of entry or endocytose in areas previously covered by it [68,69]. EM showed CCVs with HSV-1 inside after 20 min p.i. (Figure 5D). Clathrin has the ability to effectively coat vesicles destined for endocytosis of up to 200 nm in diameter [70,71]. Although the optimal size of CCVs has been described to be 100 nm [70], this is not an impediment to the entry of larger viruses such as VSV [72], cited above, or even other viruses of the *Herpesviridae* family such as the Epstein–Bar virus [73,74].

Caveolin inhibition did not appear to affect viral production in any of the trials (Figure 3A,B), which was to be expected since caveolin-lined caveolae have a diameter of 60–80 nm [75], too small to house HSV-1 virions inside. To date, caveolin-mediated endocytosis has only been described for small molecules such as certain toxins [76] or small viruses such as simian virus 40 (SV40) [77]. However, the results were not definitive, since nystatin does not selectively affect caveolin only but rather acts on the cholesterol within the cell membrane [30,31]. Lipid rafts have been shown to be used by HSV-1 for cell entry [78], consistent with the decrease in viral entry observed when nystatin was maintained for 4 h p.i. (Figure 6B). The inhibitory relationship was concentration-dependent, surely due to its effect on cholesterol. Due to the short duration of nystatin treatment [79] for protein synthesis and viral production assays (Figure 3A,B), we can only suggest that, for such exposure times—1 h p.i.—entry via caveolae was inhibited and does not appear to affect HSV-1 infection. However, increasing nystatin exposure time decreased viral entry by 50%, which seems to indicate a direct relationship between lipid-raft disorganization and decreased viral entry (Figure 6B).

Thus, we can conclude that both clathrin, through the formation of CCVs, and dynamin seem to play a role in the endocytic entry of HSV-1 into our oligodendrocytic cell line. Dynamin may also be involved in other complementary endocytic pathways that are useful for HSV-1 infection. Future studies will have to confirm the degree of involvement of all these molecules associated with endocytosis in the set of possible and feasible routes of entry of HSV-1 into its cellular host
